# A Transformer-Based Image-Guided Depth-Completion Model with Dual-Attention Fusion Module

**DOI:** 10.3390/s24196270

**Published:** 2024-09-27

**Authors:** Shuling Wang, Fengze Jiang, Xiaojin Gong

**Affiliations:** The College of Information Science and Electronic Engineering, Zhejiang University, Hangzhou 310027, China; 11831041@zju.edu.cn (S.W.); 22231079@zju.edu.cn (F.J.)

**Keywords:** depth completion, dual-attention fusion module, multi-scale dual branch

## Abstract

Depth information is crucial for perceiving three-dimensional scenes. However, depth maps captured directly by depth sensors are often incomplete and noisy, our objective in the depth-completion task is to generate dense and accurate depth maps from sparse depth inputs by fusing guidance information from corresponding color images obtained from camera sensors. To address these challenges, we introduce transformer models, which have shown great promise in the field of vision, into the task of image-guided depth completion. By leveraging the self-attention mechanism, we propose a novel network architecture that effectively meets these requirements of high accuracy and resolution in depth data. To be more specific, we design a dual-branch model with a transformer-based encoder that serializes image features into tokens step by step and extracts multi-scale pyramid features suitable for pixel-wise dense prediction tasks. Additionally, we incorporate a dual-attention fusion module to enhance the fusion between the two branches. This module combines convolution-based spatial and channel-attention mechanisms, which are adept at capturing local information, with cross-attention mechanisms that excel at capturing long-distance relationships. Our model achieves state-of-the-art performance on both the NYUv2 depth and SUN-RGBD depth datasets. Additionally, our ablation studies confirm the effectiveness of the designed modules.

## 1. Introduction

Accurate depth information is crucial for tasks such as environment perception [1], path planning [2], and obstacle detection [3]. With the rapid advancement of unmanned platform intelligence, the demands for higher accuracy and resolution in depth information are also increasing. The general approach involves using depth sensor to acquire depth information, but have significant drawbacks due to the hardware limitations, the obtained depth maps are often incomplete. Some methods try to recover accurate scene depth from noisy depth sensor data [4,5,6,7], but also has its own limitations, such as the inability to accurately determine the positions of depth edges. Based on the concurrency of edge positions in depth and color images, some approaches directly predict dense depth maps from their corresponding color image [8,9,10,11,12,13,14,15]. However, this type of method has inherent issues, such as scale ambiguity and poor generalization. In this context, our study aims to combine color images and sparse depth maps to acquire high-quality dense depth maps, known as image-guided depth-completion task. By leveraging the auxiliary structural information from color images, we can more accurately reconstruct local structures. Simultaneously, utilizing the scale information from the input depth values enhances the reliability and accuracy of obtaining dense depth maps.

Currently, the image-guided depth-completion task, which combines the use of cameras and depth sensors, remains the best choice for obtaining pixel-level scene depth maps. Meanwhile, as deep-learning methods continue to evolve in the field of computer vision, many general and effective designs have emerged, significantly advancing the task of depth completion guided by color images. The exploration of network structures has been continuous, starting from the simple encoder-decoder architectures [16], to more sophisticated two-encoder-decoder models [17,18,19], and ultimately to the dual-branch structure with two encoder-decoders [20,21,22], which have proven to yield superior depth-completion results. Additionally, spatial propagation networks [23,24,25,26,27,28] are commonly employed as post-optimization processing modules, aggregating local or non-local pixels to iteratively update the depth value of the current pixel. Furthermore, some researchers have attempted to incorporate additional spatial constraint relationships [20,29,30] or explore the network’s expressive capacity in 3D information representation [27,31,32] to enhance overall model performance.

**Figure 1:**
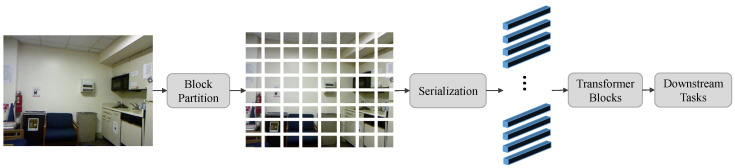
The basic framework of vision transformer.

In recent years, transformers [33] have demonstrated powerful capabilities in deep-learning tasks. They were initially designed for sequential tasks, due to their ability to capture long-range dependencies in sequences, but researchers have gradually introduced transformers to the vision domain for processing image data, leading to the proposal of the vision transformer (ViT). The ViT model has achieved impressive results in image classification [34,35,36] and object detection [35,37,38] tasks. Figure 1 shows the basic framework of ViT. It first divides an image into patches and converts them into a feature sequence. These tokens in the feature sequence are then fed into transformer blocks for processing and subsequently used for downstream tasks. However, as shown in Figure 1, this processing method embeds each image patch into a 1 × 1 feature block during the serialization process, resulting in the loss of positional information of pixels within the patch. This limitation impedes its application in dense prediction tasks.

As research on transformers has advanced, various improvements [36,39] have been proposed to adapt ViT for dense prediction tasks like semantic segmentation and monocular depth prediction. These enhancements primarily focus on obtaining high-resolution feature maps essential for accurate dense predictions. Building on this, researchers [22,40] have begun exploring the integration of vision transformers into color image-guided depth-completion tasks. GuideFormer [22] employs a fully transformer-based architecture for dense depth completion. It processes sparse depth and color guidance images through separate transformer branches to extract complementary token representations. The paper also introduces an effective token-fusion method using a guided-attention mechanism to explicitly model information flow between the two branches and capture intermodal dependencies. CompletionFormer [40] proposes a joint convolutional attention and transformer block (JCAT), which integrates convolutional attention and vision transformer layers into a single block. This unit is used to construct the depth-completion model in a pyramidal structure.

In this study, we design an image-guided depth-completion model that leverages advancements in transformers by thoroughly analyzing the strengths and limitations of existing methods. Specifically, we adopt an effective dual-branch network structure comprising dual encoders and dual decoders. Both encoders use transformer-based modules as the core component to extract features from the input data. The decoders, in turn, employ deconvolution operations to progressively recover feature resolutions and generate corresponding depth maps. While the plain ViT [22,39] divides the image into patches only once, leading to single-scale features with lower resolution, our model enhances this approach. We divide the image and its features into patches at multiple stages, embed these patches into tokens, and feed them into transformer blocks. This strategy allows us to capture feature relationships across tokens and generate multi-scale pyramid features at various stages, which are more effective for dense prediction tasks.

The two branches of the dual-branch model are not independent but involve additional information interaction. In addition to integrating the depth maps output by each branch, our model uses the output of the first branch as the input for the second branch [21], and design a dual-attention fusion module to achieve decoder-encoder fusion, enhancing the connection between the two branches. This module fully utilizes two types of attention mechanisms: convolution-based attention, which captures local information within corresponding regions, and transformer-based cross-attention, which captures long-range correlations. A cross-attention module processes two different inputs, with distinct configurations of Q, K, and V, representing different physical meanings. Therefore, we thoroughly investigate and analyze these configurations and explore their implications on the final outcomes in the experiment section.

The main contributions of this study are summarized as follows:

1. We apply transformer technology to the color image-guided depth-completion task by designing a dual-branch network structure. This structure divides the image and its features into patches at multiple steps, and generates multi-scale pyramid features to better adapt to the characteristics of dense prediction tasks.

2. We construct a dual-attention fusion module, incorporating spatial and channel-attention mechanisms and cross-attention mechanisms to strengthen the decoder-encoder fusion between the two branches. Additionally, it explores the impact of different configurations of Q,K, and V in the cross-attention mechanism on depth results.

3. We validate the effectiveness of our network through relevant experiments on the NYUV2 [41] and the SUN-RGBD datasets [42].

## 2. Related Work

### 2.1. Vision Transformer

Transformers excel in modeling long-range dependencies and possess a global receptive field, making them effective in various fields such as image classification [34,35,36] and object detection [35,37,38], where they have achieved notable results. In these applications, transformers typically function as encoders to extract image features, which are then fed into task-specific decoders. However, the original vision transformer (ViT) produces single-scale, low-resolution feature maps, which are inadequate for dense prediction tasks that require high-resolution features. Therefore, improvements on transformer models are necessary for their effective use in dense prediction tasks. Ranftl et al. [39] address this issue by assembling tokens from various stages of the vision transformer into image-like representations at multiple resolutions, progressively combining them into full-resolution predictions using a convolutional decoder. Similarly, Wang et al. [36] introduce a spatial-reduction attention mechanism, constructing a progressively shrinking feature pyramid structure, which enables the model to flexibly learn multi-scale and high-resolution features. The Swin transformer [35] achieves multi-scale feature extraction through alternating stages of shifting windows and non-overlapping patch partitioning. This hierarchical approach, which merges varying numbers of image feature patches to construct feature maps of different scales and performs self-attention calculations within local windows, facilitates effective feature extraction and modeling capabilities at multiple scales.

### 2.2. CNN and Transformer

**Figure 2:**
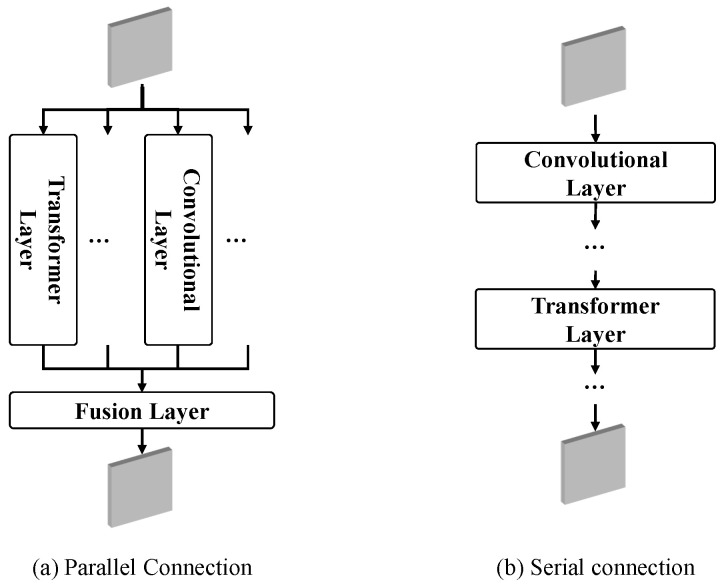
The combination of transformer layers and convolutional layers.

Vision transformers have been successfully applied to vision tasks due to their ability to capture long-range dependencies within an image. However, there are still gaps in both performance and computational cost between transformers and existing convolutional neural networks (CNNs), due to their deteriorated local feature details. To address this issue, as shown in Figure 2, many current studies integrate the structures of transformers and convolutional layers either in parallel [37,43] or in series [44].

Guo et al. [44] introduce a novel hybrid network that combines the transformer’s ability to capture long-range dependencies with the local feature extraction capabilities of CNNs in a sequential manner. This approach achieves a better balance between accuracy and efficiency. Similarly, Lite-Mono [43] adopts a sequential structure, featuring a continuous dilated convolution module for extracting rich multi-scale local features, and a local-global feature interaction module that uses the self-attention mechanism to encode long-range global information into the local features. MPViT [37] uses overlapping convolutional patch embedding to simultaneously embed features of the same size with patches of different scales, enabling fine and coarse feature representations at the same feature level. Additionally, joint convolutional attention and transformer (JCAT) [40] combines convolutional attention layers and self-attention modules to create the basic unit of a depth-completion model, proposing both parallel and serial architectures. These approaches demonstrate that integrating the strengths of CNNs and Transformers can address the limitations of each method when used alone, leading to more efficient and accurate models for various visual tasks.

### 2.3. Depth-Completion Network Architecture Design

Many studies have developed specialized network architectures for the image-guided depth-completion task. A direct method uses a traditional encoder-decoder network to address the pixel-to-pixel regression problem [16] by concatenating the image with the sparse depth or their embeddings at the network’s input. Dual-encoder single-decoder networks, on the other hand, learn domain-specific features from RGB images and sparse depth maps using two separate encoders, which are then fused to create a correlated feature representation within a single decoder [17,18,19]. The dual-encoder dual-decoder network [45,46,47] enhances this approach by introducing an additional decoder. In this architecture, two encoder-decoder networks process the image and sparse depth inputs separately. The high-resolution features from the image encoder-decoder network are then utilized to guide the depth recovery process of the sparse depth decoder.

The core design principle of the dual-branch structure is to predict depth maps from two complementary perspectives, such as global and local views [48], or from color images and depth maps [21,22,49]. The predictions from these two branches are then adaptively integrated. Two-stage networks often employ a coarse-to-fine prediction strategy to enhance depth accuracy. In the coarse stage, an initial dense depth map is generated, which is then refined in the fine stage by integrating color images to produce a more precise output. Post-refinement modules are primarily based on spatial propagation network (SPN) methods [23,24,25,26,27,28]. These methods first generate a dense but coarse depth map and then iteratively refine it by aggregating neighboring pixel points using input-adaptive guided kernels.

#### Depth Completion Based on Transformer

As transformer applications in dense prediction tasks gain prominence, research has increasingly integrated transformers into color image-guided depth-completion tasks. GuideFormer [22] employs a dual-branch architecture entirely based on transformers, with one branch processing sparse depth and the other handling color images. To fully utilize the guiding role of color images, Feng et al. [50] introduce multi-layer cross-attention modules to integrate features from depth and color images at various scales. PointDC [32] is a feature point cloud aggregation framework that directly propagates 3D depth information between given and missing points. This method first extracts 2D features from images, converts sparse depth maps into point clouds to obtain sparse 3D features, and then aggregates neighboring sparse 3D features of reference pixels using cross-attention mechanisms to reconstruct depth information at target locations.

## 3. Method

**Figure 3:**
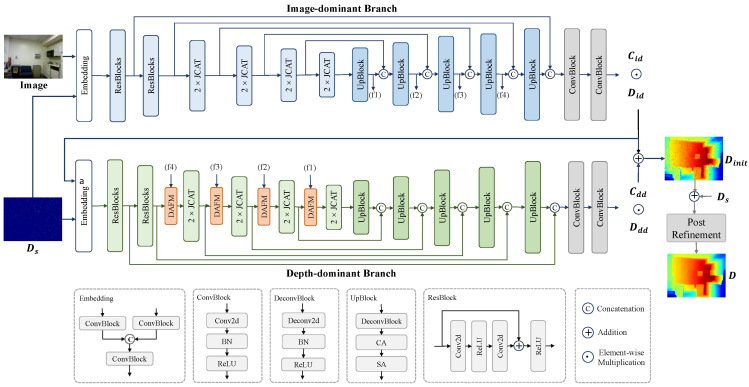
An overview of our framework: a dual-branch backbone network consisting of an image-dominant branch and a depth-dominant branch, along with a post-refinement module. In the framework, Image represents the input color image and $D_{s}$ denotes the sparse depth map input to the network. $D_{id}$ and $C_{id}$ represent the depth map and its corresponding confidence map output from the image-dominant branch, while $D_{dd}$ and $C_{dd}$ correspond to the depth map and confidence map from the depth-dominant branch. $D_{init}$ is the depth map obtained from the fusion of the two branches, which is the output of the backbone, and D represents the final depth map after post-refinement, which is the output of the entire network.

Figure 3 illustrates the overall architecture of the color image-guided depth-completion network. Our model comprises two main components: a dual-branch backbone network and a post-processing optimization module based on a spatial propagation network (SPN). Both branches use an encoder-decoder structure, featuring a decoder-encoder fusion module to enhance the connection between the branches. The following sections will provide a detailed explanation of each part of the network, including the transformer-based pyramid dual-branch backbone network, the decoder-encoder dual-attention fusion module, the SPN-based post-processing optimization module, and the loss function.

### 3.1. Transformer-Based Pyramid Dual-Branch Backbone

The dual-branch backbone network includes an image-dominant branch and a depth-dominant branch, both utilizing a similar encoder-decoder structure. The encoder employs transformer-based modules, leveraging their robust feature extraction capabilities to encode input data, while the decoder uses transposed convolution-based modules to gradually recover feature scales. We avoid using transformer-based modules throughout the entire structure to prevent unnecessary resource consumption and ensure that each module serves its intended function within the model. To address the limitations of single-scale and low-resolution features in plain ViT [22,39], our model progressively divides the image or feature map into patches step by step. At each step, these patches are serialized into tokens. Transformer-based blocks are then used to capture the relationships between these tokens, generating multi-scale pyramid features. The following sections will detail the specific structure of each part of the network.

#### 3.1.1. Image-Dominant Branch

The color variations in the image closely align with depth value changes in the depth map, enabling the image to provide critical edge and detail information for depth prediction. Therefore, we design the image-dominant branch, which primarily relies on color information to predict depth map. To improve effectiveness, an aligned sparse depth map is also incorporated as input to assist in the depth prediction, providing scale information that facilitates the final fusion of the two branches.

As depicted in the “Embedding” module in Figure 3, we first use two separate ConvBlocks to encode the input sparse depth map and color image. The outputs are then concatenated and processed by another ConvBlock to obtain features that incorporate information from both inputs. Each ConvBlock consists of a 2D convolution layer, a batch normalization (BN) layer, and a ReLU activation layer [51], as shown in the “ConvBlock” module in Figure 3. The “Embedding” module allows the feature vector of each pixel to simultaneously contain both image and depth information, enabling the correction of invalid depth pixels through reliable depth measurements based on visual similarity. Following this, we utilize the initial two layers from ResNet34 [52], specifically some ResBlock layers, to obtain features at full resolution and half resolution, denoted as $F^{1}\in R^{H\times W\times C^{1}}$ and $F^{2}\in R^{H/2\times W/2\times C^{2}}$, respectively.

The subsequent four layers incorporate transformer-based modules as the fundamental units of the framework design. At the beginning of each layer, a patch embedding module serializes the features. This module first divides the feature map $F^{i-1}$ from the previous stage i−1 into patches of size 2×2. This is achieved using a convolution layer with a kernel size of 3×3 and a stride set to 2, effectively halving the resolution of the feature map $F^{i-1}$. These four layers produce the multi-scale feature pyramid necessary for the task, denoted as $F^{3}$, $F^{4}$, $F^{5}$, and $F^{6}$, where $F^{i}\in R^{H/2^{i-1}\times W/2^{i-1}\times C^{i}}$. The output from the patch embedding in each layer is then fed into a joint convolutional attention and transformer (JCAT) module [40], which processes these multi-scale pyramid features.

The decoder progressively recovers feature scales using five UpBlock layers. Each UpBlock layer consists of a DeconvBlock for upsampling features to the current scale, incorporating a convolutional attention module [53]. The DeconvBlock includes a transposed 2D convolution, a batch normalization layer, and a ReLU activation function, as illustrated in Figure 3. Additionally, skip connections are employed during scale recovery to integrate features from corresponding scales in the encoder. The input to the first UpBlock layer in the decoder is the final layer of features from the encoder, $F^{6}$. The inputs to the subsequent four UpBlock layers consist of the outputs from the previous layer concatenated with the corresponding scale features from the encoder, namely $F^{5}$, $F^{4}$, $F^{3}$, and $F^{2}$. The final output layer also incorporates the full-resolution features $F^{1}$ as inputs. This layer comprises two ConvBlocks, with the last one producing two output channels corresponding to the depth map $D_{id}$ and the confidence map $C_{id}$.

#### 3.1.2. Depth-Dominant Branch

In addition to the information from the color image, our input also includes a sparse depth map, which shares the same modality as the output depth map. Therefore, we construct the depth-dominant branch to leverage the available depth information for depth prediction. The depth-dominant branch takes only depth modality inputs, including the sparse depth map and the depth map $D_{id}$ produced by the image-dominant branch as prior depth information.

This branch shares a similar structure with the image-dominant branch. As depicted in Figure 3, these inputs undergo initial fusion through an embedding layer before being fed into an encoder similar to that of the image-dominant branch. However, the depth-dominant branch incorporates an additional dual-attention fusion module (DAFM) in its encoder to merge features from corresponding scales in the image-dominant branch’s decoder, enhancing the fusion between the two branches [21,31]. The design details of the DAFM will be elaborated in the next section. The decoder part of this branch follows the same structure as the image-dominant branch and outputs the corresponding depth map $D_{dd}$ and confidence map $C_{dd}$ in the final output layer.

#### 3.1.3. Dual-Branch Fusion

Finally, as described in Equation (1) [21,22,48], the backbone network uses the confidence maps $C_{id}$ and $C_{dd}$ from the two branches to combine the depth maps $D_{id}$ and $D_{dd}$ produced by each branch, resulting in the final depth map.

(1) $$D_{init}=\frac{C_{id}D_{id}+C_{dd}D_{dd}}{C_{id}+C_{dd}}$$

Here, $D_{init}$ represents the output of the dual-branch backbone network. Dividing by $C_{id}+C_{dd}$ ensures that the values of $D_{init}$ remain within a reasonable range. This weighted normalization approach effectively fuses the depth maps produced by the two branches.

### 3.2. Dual-Attention Fusion Module

**Figure 4:**
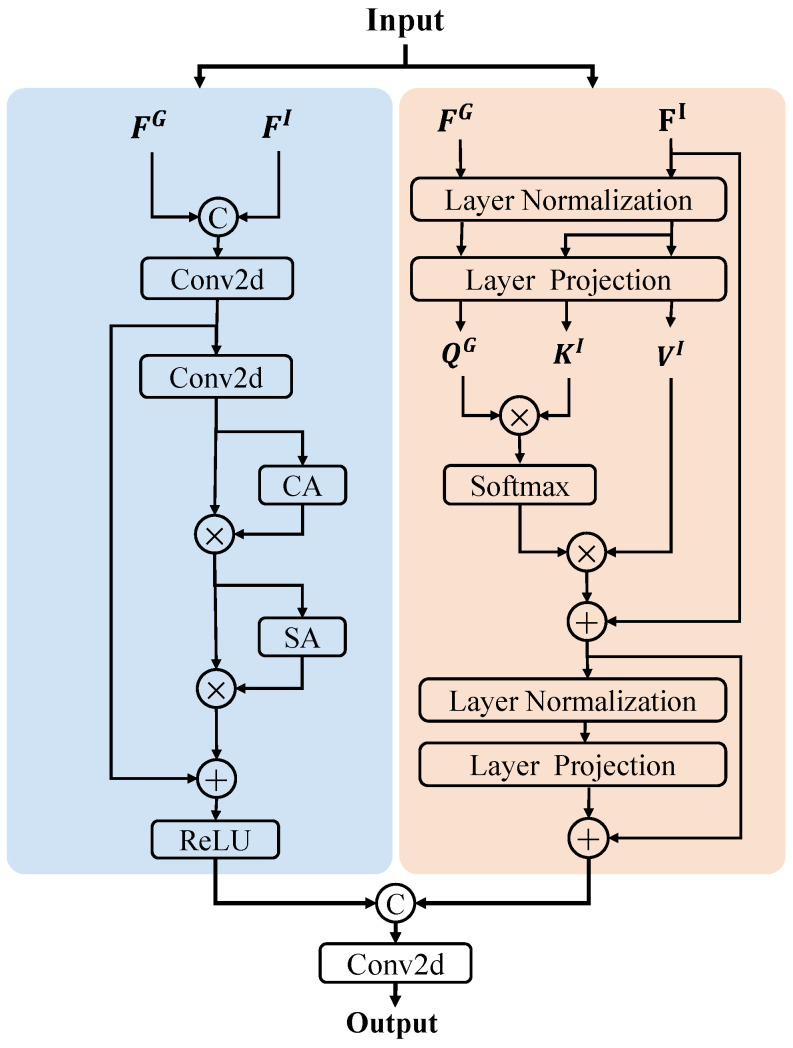
The Framework of the dual-attention fusion module.

The dual-attention fusion module (DAFM) enhances the integration of input features $F^{I}\in R^{H^{i}\times W^{i}\times C^{i}}$ from the depth-guided branch encoder and the guidance features $F^{G}\in R^{H^{i}\times W^{i}\times C^{i}}$ from image-guided branch decoder across multiple layers. As depicted in Figure 4, this module comprises two branches: a transformer-based cross-attention mechanism excels at capturing long-distance relationships, while a convolution-based attention mechanism prioritizes local details. This integration creates a unified framework that effectively captures both global relationships and fine-grained details.

#### 3.2.1. Transformer-Based Cross-Attention

In the cross-attention mechanism, different configurations of Q, K, and V denote distinct physical meanings. In the context of depth-completion tasks, the transformer-based cross-attention branch aims to utilize multi-scale features from the color image-dominant branch as guiding features $F^{G}$ to enhance feature extraction from corresponding scale input features $F^{I}$ in the depth-dominant branch encoder. To achieve this, we designate the guiding features and input features as queries (Q) and keys (K) to generate a correlation matrix, using the input features as values (V) to enhances the features of $F^{I}$.

As shown in Figure 4, both input features $F^{I}\in R^{H^{i}\times W^{i}\times C^{i}}$ and guiding features $F^{G}\in R^{H^{i}\times W^{i}\times C^{i}}$ are first processed through layer normalization (LN) and then through their respective layer projection (LP) layers $W^{q}$, $W^{k}$ and $W^{v}$. The input features $F^{I}$ are passed through the $W^{k}$ and $W^{v}$ layers to produce $K^{I}\in R^{N\times C^{i}}$ and $V^{I}\in R^{N\times C^{i}}$, respectively. Meanwhile, the guiding features $F^{G}$ are processed through the $W^{q}$ layer to obtain $Q^{G}\in R^{N^{i}\times C^{i}}$, where $N^{i}=\frac{H^{i}}{2}\times \frac{W^{i}}{2}$. The projection layers use convolutional operations to flatten and serialize the feature maps into tokens. To preserve the positional information of each token in the original image, each token is combined with learnable positional embeddings ${\mathrm{pe}}^{I}\in R^{H^{i}\times C^{i}}$ and ${\mathrm{pe}}^{G}\in R^{N^{i}\times C^{i}}$, as described in Equation (2).

(2) $$Q^{G}=Q^{G}+{\mathrm{pe}}^{G},\;K^{I}=K^{I}+{\mathrm{pe}}^{I},\;V^{I}=V^{I}+{\mathrm{pe}}^{I}$$

Subsequently, $Q^{G}$, $K^{I}$ and $V^{I}$ are fed into an attention function [33] as follows:

(3) $$Attn\left(Q^{G},K^{I},V^{I}\right)=softmax\left(\frac{Q^{G}{\left(K^{I}\right)}^{T}}{\sqrt{d}}\right)V^{I}$$

This operation computes a weighted sum of the values $V^{I}$, where the attention scores, determined by the similarity between the query $Q^{G}$ (projected guiding features) and the key $K^{I}$ (projected input features), dictate the contribution of each value. The channel dimension of the query is denoted by *d*. The scaling factor $\sqrt{d}$ is introduced to prevent the dot product values from becoming excessively large. Finally, the output features of this branch are obtained after applying layer normalization and a projection layer $W^{p}$, with residual connections.

According to Equation (3), each token in the input feature set is matched with tokens from all positions in the guiding features, including those in corresponding positions. This process effectively expands the receptive field to cover the entire image. Since each token in the input features contains explicit depth information and each token in the guiding features embeds dual information from both the image and depth, this attention mechanism explicitly compares each token, i.e., each image patch’s similarity, through the dot product operation. This similarity-based guidance allows reliable depth information to propagate throughout the entire image, thereby enhancing the depth features.

#### 3.2.2. Convolution-Based Attention

The transformer-based cross-attention branch focuses on exploring correspondences between different patches. In contrast, the convolution-based attention branch models local features at corresponding positions more precisely, utilizing both channel and spatial attention mechanisms. This approach ensures accurate representation of local details in the features.

As shown in Figure 4, the input feature $F^{I}$ and guiding feature $F^{G}$ are first concatenated and then processed through a convolution layer for preliminary feature extraction. The fused features are then further refined using the channel attention (CA) and spatial attention (SA) modules [53]. The CA module creates a channel attention map based on inter-channel relationships, helping to identify the significant features across channels. This module focuses on determining “what” features are important. Complementarily, the SA module generates a spatial attention map by considering spatial relationships within the features, focusing on “where” the informative regions are located. Together, these modules enhance the feature representation by addressing both channel and spatial aspects.

In the final stage of the module, the output from the transformer-based cross-attention branch, $F^{T}\in R^{H/2\times W/2\times C}$, is concatenated with the output from the convolution-based attention branch, $F^{C}\in R^{H/2\times W/2\times C}$. This concatenated feature is then processed through a convolution layer with a kernel size of 3. The resulting output, which represents the fused features from both branches, is then passed to the next stage of the network.

### 3.3. Post Refinement Module

The depth values of initially valid points in the sparse map may be changed and not properly maintained by the backbone network. Simply replacing these values with the original depths could result in inconsistencies with the surrounding regions. To address this issue, we introduce a post-processing module based on spatial propagation networks, which enhances the model’s performance by better preserving depth consistency.

Specifically, we employ the non-local spatial propagation network (NLSPN) [25] as the post-processing optimization module. Let $Dt=\left(d_{u,v}^{t}\right)\in R^{H\times W}$ denote the depth map obtained in the *t*-th iteration of propagation, where $d_{u,v}^{t}$ represents the depth value at pixel (u,v), and *H* and *W* denote the height and width of the depth map, respectively. The propagation relationship between $d^{t}u,v$ and its non-local neighborhood $N_{u,v}^{NL}$ in the *t*-th iteration is defined as follows, according to Equation (4):

(4) $$d_{u,v}^{t}=w_{u,v}\left(0,0\right)d_{u,v}^{t-1}+\sum_{\left(i,j\right)\in N_{u,v}^{NL},i\neq 0,j\neq 0}w_{u,v}\left(i,j\right)d_{i,j}^{t-1}$$

where $w_{u,v}\left(i,j\right)$ represents the correlation weight between the reference pixel (u,v) and pixel (i,j) in its neighborhood $N_{u,v}$. The term $w_{u,v}\left(0,0\right)=1-\sum_{\left(i,j\right)\in N_{u,v},i\neq 0,j\neq 0}w_{u,v}\left(i,j\right)$ denotes the proportion of the original depth value $d_{u,v}^{t-1}$ that is preserved.

The correlation weights w are learned by the network and modulated by the confidence map predicted by the network. The confidence map helps avoid spatial propagation on pixels with high correlation but low confidence. The model employs NLSPN in a residual manner, where the depth map output by the backbone network is combined with the original sparse depth map. This combined input, denoted as $D_{init}+D_{sd}$, is used for further processing.

### 3.4. Loss Function

We employ a combined $L_{1}$ and $L_{2}$ loss function for the depth map. The loss function is defined as follows in Equation (5):

(5) $$\begin{array}{cc} L\left(D,D_{gt}\right)=\frac{1}{N}( & {∥1\left(D_{gt}>0\right)⊙\left(D-D_{gt}\right)∥}_{2}\\ + & {∥1\left(D_{gt}>0\right)⊙\left(D-D_{gt}\right)∥}_{1}) \end{array}$$

where D represents the network’s predicted depth map and $D_{gt}$ denotes the ground truth depth map used for supervision. ${∥\cdot ∥}_{2}$ represents the $L_{2}$ norm. 1 is an indicator function that takes the value of 1 when the ground truth depth map is greater than 0, and 0 otherwise. This approach supervises only the pixels in the depth map where depth values are valid.

As described earlier in the network structure, the entire architecture involves the output of four depth maps: the final predicted depth map D, the initial depth $D_{init}$ output by the backbone network, the depth map $D_{id}$ output by the color image dominant branch, and the depth map $D_{dd}$ output by the depth dominant branch. Constraints are applied on all four depth map outputs to ensure stable training of the network. Therefore, the overall network loss function can be defined as Equation (6):

(6) $$L=\lambda L\left(D,D_{gt}\right)+\lambda_{init}L\left(D_{init},D_{gt}\right)+\lambda_{id}L\left(D_{id},D_{gt}\right)+\lambda_{dd}L\left(D_{dd},D_{gt}\right)$$

where λ, $\lambda_{init}$, $\lambda_{id}$, and $\lambda_{dd}$ represent the weights assigned to D, $D_{init}$, $D_{id}$, and $D_{dd}$, respectively.

## 4. Experiment

### 4.1. Experiment Setting

In this section, we will introduce the datasets used to validate the effectiveness of the method, the evaluation metrics, and the relevant settings employed during model training.

#### 4.1.1. Dataset

**NYUv2** The NYUv2 dataset [41] contains video sequences of 464 indoor scenes recorded with a Microsoft Kinect depth sensor, resulting in a total of 407,024 pairs of color images and corresponding raw depth maps, each with a resolution of 640 × 480. Following the settings of previous depth-completion methods [25,40,48], we train our model on the official training set, which contains 249 scenes and 50,000 pairs of uniformly sampled images, and test it on the official test set, which contains 215 scenes and 654 pairs of images. The groundtruth depth maps are derived from the raw depth data using the method provided in the official toolbox. For algorithm implementation, the original images are downsampled to 320×240, then center-cropped to 304×228 for both training and testing. The sparse depth maps used for these processes are generated by randomly sampling from the dense ground truth depth maps.

**SUN RGB-D** The SUN RGB-D dataset [42] contains 10,335 RGB-D images captured by four different sensors. Following the official split, we used 5285 images for training and 5050 images for testing, covering 19 major scene categories. The refined depth maps, based on multiple frames integration, were used as groundtruth depth maps. For algorithm implementation, The input images were resized to 320×240 and center-cropped to 304×228 for both training and testing.

#### 4.1.2. Evaluation Metrics

For evaluating the predicted depth map *D* against the ground truth depth map $D_{gt}$, we use several metrics. For the ablation study, we use five metrics: root mean square error (RMSE), mean absolute error (MAE), inverse RMSE (iRMSE), inverse MAE (iMAE), and absolute relative error (REL). For comparison with other methods on the NYUv2 dataset, we use commonly used benchmark metrics: RMSE, REL, and the percentage of pixels within thresholds $\delta_{1.25}$, $\delta_{1.25^{2}}$, and $\delta_{1.25^{3}}$. For the SUN RGB-D dataset, we use benchmark metrics including RMSE, MAE, REL, Structural Similarity Index Measure (SSIM), and the percentage of pixels within the threshold $\delta_{1.05}$.

#### 4.1.3. Implementation Details

We implement our model in PyTorch. The AdamW optimizer is employed with an initial learning rate of 0.001, β1 = 0.9, β2 = 0.999, and weight decay of 0.01. The model is trained 100 epochs, with a learning rate decay of 0.5 at epochs 36, 48, 60, 72, 80, and 88. For the NYUv2 dataset, we train the model using four NVIDIA 3090 GPUs with a batch size of 32. For the SUN RGB-D dataset, we use a single GPU with a batch size of 8. For hyper-parameters $\lambda_{init}$, $\lambda_{id}$, and $\lambda_{dd}$: Epoch<25, $\lambda_{id}$, $\lambda_{dd}$ = 0.2, $\lambda_{init}=1.0$. 25≤Epoch<50, $\lambda_{id}$, $\lambda_{dd}=0$, $\lambda_{init}=0.5$. Epoch≥50, $\lambda_{init}=0$.

### 4.2. Visualization

**Figure 5:**
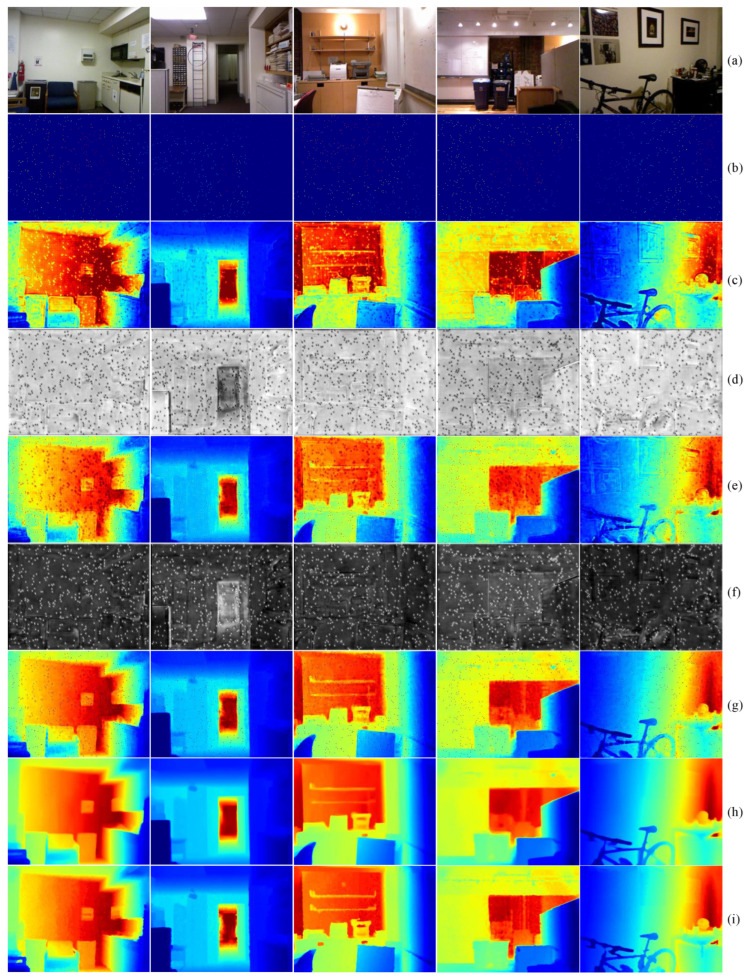
Visualization of intermediate results in NYUv2 Dataset: (**a**) RGB image; (**b**) sparse depth; (**c**) depth from image branch; (**d**) confidence map from image branch; (**e**) depth from depth branch; (**f**) confidence map from depth branch; (**g**) depth from the dual-branch backbone; (**h**) final depth result of the model; and (**i**) groundtruth.

In Figure 5, we visualize the intermediate variables and final depth maps produced by our model on the NYUv2 dataset to better understand the function of each component. From Figure 5c,e, we observe that the depth maps generated by the two branches of the backbone network can generally restore the basic 3D structure of the scene. The corresponding confidence maps reveal that the image-dominated branch typically assigns higher confidence to edge areas, as seen in the results for all four scenes. In the depth-dominated branch, higher confidence is noted in distant areas, particularly in scenes with a pronounced depth of field. For instance, in the second scene, the confidence level is notably higher behind the door than in other areas. It is noted that speckled patterns may appear due to the inherently high sparsity of the depth map and the incorporation of sparse depth data before the post-processing module. Nevertheless, the final depth prediction of the network shows smooth transitions and variations in depth values.

### 4.3. Ablation Study

#### 4.3.1. Mulit-Scale Dual-Branch Framework

The experiment in this section evaluates the effectiveness of the dual-branch design, multi-scale pyramid features, and post-processing optimization modules. In Table 1, “Single-B” refers to a single-branch structure, while “Dual-B” denotes a dual-branch structure. In the “Pyramid” column, ✓ indicates the use of multi-scale pyramid structures, whereas entries without ✓ indicate the use of only a single scale (1/8) of features. Similarly, in the “NLSPN” column, ✓ signifies the inclusion of a post-processing module.

By comparing the first and third rows of the table, significant advantages of the dual-branch structure over the original single-branch network can be observed. The comparison between the second and third rows demonstrates a clear advantage of multi-scale pyramid features over single-scale features. Lastly, comparing the results between the third and fourth rows shows substantial improvements in evaluation metrics after adding NLSPN, particularly notable in the improvement of MAE.

#### 4.3.2. Loss Function

In this section, we conducted experiments on the hyper-parameters of the weights λ, $\lambda_{init}$, $\lambda_{id}$, and $\lambda_{dd}$ assigned to the predicted depth maps D, $D_{init}$, $D_{id}$, and $D_{dd}$ in the loss function, and the results are presented in Table 2 below.

Table 2 presents the results under three parameter settings. In the first setting, we only apply constraints to the final depth output D of the network, meaning that throughout the training process, λ is set to 1, while all other weights $\lambda_{init}$, $\lambda_{id}$, $\lambda_{dd}$ are 0. In the second setting, we apply constraints to all depth outputs of the network, but the values remain constant throughout the training processs. In the final setting, constraints are applied to all parameters at the beginning of training, and as training progresses, the supervision weights for intermediate depth predictions gradually decrease, eventually applying constraints only to the network’s final depth output D. From the experimental results, it can be observed that the third setting achieved the best results.

#### 4.3.3. Dual-Attention Fusion Module

In this section, we validate the effectiveness of various designs of the dual-attention fusion module. To enhance the efficiency of the ablation study, the experimental model presented in this section incorporates only a dual-branch backbone network, excluding the NLSPN module.

**The effectiveness of dual attention:** We analyze the effectiveness of fusing two types of features by integrating convolution-based attention and transformer-based cross-attention. As shown in Table 3, “Conv” and “Trans” represent the two corresponding attention mechanism branches, and the ✓ in each column indicates whether that branch is used in the module. The comparison results demonstrate that the combined dual-attention fusion module outperforms each individual attention mechanism, whether convolution-based attention or transformer-based cross-attention.

**The effectiveness of the Q,K,V configuration in cross-attention**: This subsection explores the physical meanings of different settings for Q, K, and V in cross-attention mechanisms, and analyzes their impact on results as shown in Table 4. Specifically, $Q^{G}$, $K^{G}$, $V^{G}$ denote Q, K, V generated using guided feature $F^{G}$, while $Q^{I}$, $K^{I}$, $V^{I}$ represent that generated using input feature $F^{I}$.

**Figure 6:**
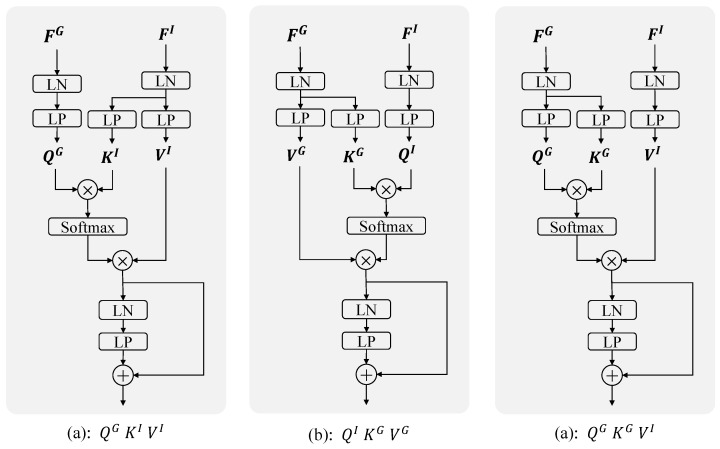
The schematic diagram of cross-attention with different configurations of Q,K,V.

In typical attention mechanisms, Q and K are initially multiplied to compute a correlation matrix, which is subsequently used to weight V for aggregating global information. Following this, Figure 6 illustrates different configurations of Q, K, and V in cross-attention.

- $Q^{G},K^{I},V^{I}$: Use the guided feature and input feature as $Q^{G}$ and $K^{I}$, respectively, to generate the correlation matrix, aggregating the information of input feature $V^{I}$.
- $Q^{I},K^{G},V^{G}$: Use the guided feature and input feature as $Q^{I}$ and $K^{G}$, respectively, to generate the correlation matrix, aggregating the information of feature $V^{G}$.
- $Q^{G},K^{G},V^{I}$: Use the guided feature simultaneously as $Q^{G}$ and $K^{G}$ to generate the correlation matrix, aggregating the information of input feature $V^{I}$.

From the results in Table 4, there are no significant differences among the outcomes of the three configurations. All configurations represent reasonable approaches for this task. However, overall, the configuration with $Q^{G}$, $K^{I}$, and $V^{I}$ demonstrates better performance compared to the others.

### 4.4. Comparision with SOTA

**NYUv2 dataset:** Table 5 presents a comparison of our method with the current state-of-the-art techniques on the NYUv2 dataset. The notation Ours* refers to the results obtained with the dual-branch backbone network, excluding the post-processing optimization module NLSPN, while Ours denotes the complete model with NLSPN. Even without the NLSPN module, Ours* achieves performance comparable to the state-of-the-art methods. After incorporating the NLSPN module, Ours shows a significant breakthrough in the REL metric and achieves state-of-the-art performance across all metrics.

**Figure 7:**
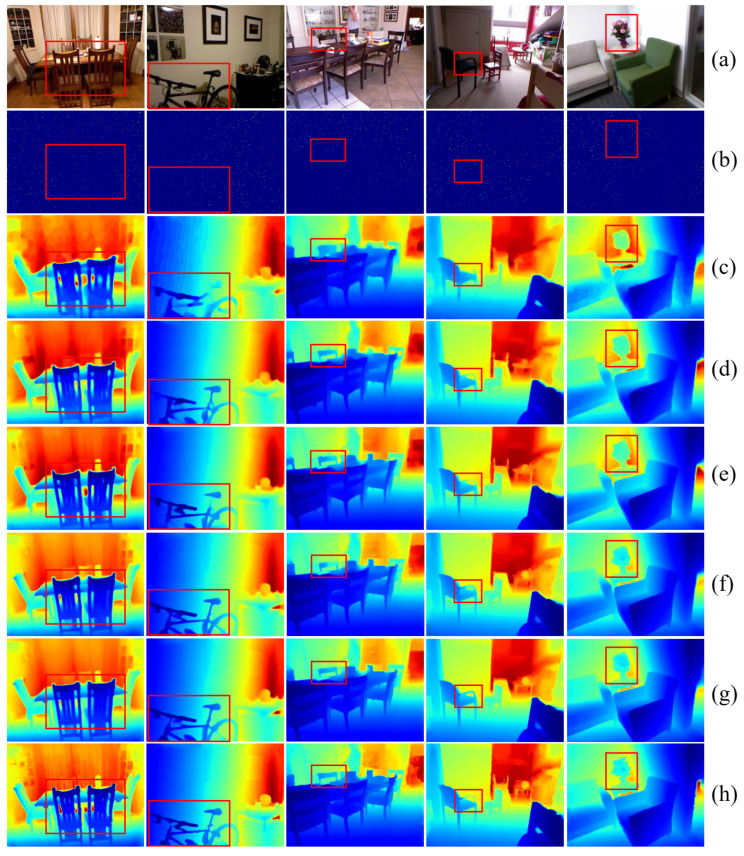
Visualization comparison with mainstream methods: (**a**) color image; (**b**) sparse depth map; (**c**) predicted depth map by CSPN [23]; (**d**) predicted depth map by CostDCNet [57]; (**e**) predicted depth map by NLSPN [25]; (**f**) predicted depth map by CompletionFormer [40]; (**g**) Ours; (**h**) groundtruth depth map.

We also tested several methods that provided training models [23,25,40,57], obtained their corresponding predicted depth maps, and compared them with the predictive results of our method. The results are shown in Figure 7. From the experimental results, our method demonstrates a smoother overall depth variation and sharper depth changes in edge areas visually.

For instance, in the first scene, the backrest of the chair is more distinctly differentiated from the background in our method compared to all other methods. Similarly, in the second scene, the silhouette of the foreground bicycle frame is clearer and more prominent. Moreover, the depth variation within local objects is more coherent and smooth. For example, in the third scene, the backrest of the chair shows a smoother depth variation and clearer outline in our results compared to CompletionFormer [40]. The same situation occurs in the fourth scene with the thin rods at the edge of the chair. Additionally, the depth variation within object areas is more accurate. For instance, in the fifth scene with the bouquet of flowers, other methods mostly predict it as a whole without depth variation, while only our method and CompletionFormer [40] can accurately predict the internal depth variations.

**SUN-RGBD dataset:** Table 6 presents a comparison between our method and other approaches on the SUN-RGBD dataset. In this benchmark, the input depth maps for completion are raw images from depth sensors. For a fair comparison, we evaluated under the same input settings. The results show that while our method performs slightly worse in SSIM, it exhibits clear advantages in RMSE, MAE, REL, and $\delta_{1.05}$.

**Figure 8:**
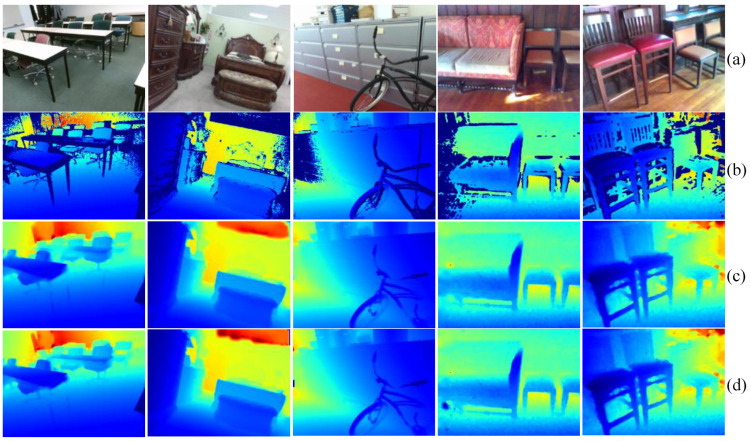
Visualization of completion results of raw depths which are directly captured by depth sensor in the SUN-RGBD dataset: (**a**) RGB image; (**b**) raw depth; (**c**) final depth result of the model; and (**d**) groundtruth.

**Figure 9:**
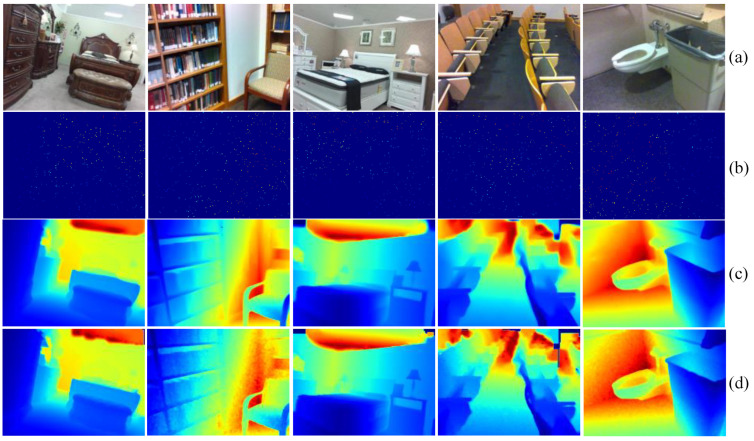
Visualization of completion results of sparse raw depths which are generated by randomly sampling from the raw depth maps in the SUN-RGBD dataset: (**a**) RGB image; (**b**) sparse raw depth; (**c**) final depth result of the model; and (**d**) groundtruth.

We present the visualization results on the SUN-RGBD dataset in Figure 8. The results show that, even with just over 5000 training samples, our model can accurately recover scene information. Additionally, we conducted an extra completion experiment using sparse raw depth maps on this dataset, where the sparse depth maps were generated by randomly sampling from the raw depth maps as shown in Figure 9. The results indicate that, even with only a few sparse points as input, our model effectively recovers fine-grained structural details of the scene.

### 4.5. Model Inference Efficiency Analysis

In this section, we analyze the inference efficiency of different model variants, including inference time, memory usage, model size, and final performance (RMSE). These metrics highlight the trade-offs between accuracy and computational cost for each model component. Specifically, we present the results for three variants: the single-branch model (Single-B), the dual-branch model (Dual-B), and the dual-branch model with the NLSPN post-processing module(Dual-B + NLSPN) in Table 7.

From the results in the Table 7, we can see that, while the dual-branch model increases computational cost to some extent compared to the single-branch model, the performance improvement is significant. The NLSPN post-processing module adds minimal parameters but results in higher inference time and memory usage due to its iterative convolution operations along the time dimension. However, it brings only a slight improvement in RMSE. Based on Table 7, we can choose different models depending on the practical application requirements.

## 5. Conclusions

In this paper, we design an effective image-guided depth-completion model, proposing a dual-branch network architecture backbone with attention mechanisms. The approach begins with a phased image feature tokenization strategy to extract multi-scale pyramid features for pixel-wise dense prediction tasks. Additionally, a dual-attention fusion module is designed to integrate decoder-encoder fusion between the two branches, combining convolution-based attention mechanisms with transformer-based cross-attention mechanisms to capture both long-range dependencies and preserve local details. Finally, experimental results on the NYUv2 depth dataset and SUN-RGBD dataset validate the effectiveness of the proposed model and its modules. This method not only significantly improves the accuracy of depth-completion tasks but also offers new perspectives and tools for further applications of deep learning in image processing.

## Figures and Tables

**Table 1:** Multi-scale dual-branch framework.

Model	Pyramid	NLSPN	RMSE	MAE	iRMSE	iMAE	REL
Single-B	✓		0.108	49.94	17.77	7.82	0.017
Dual-B			0.094	38.79	14.83	5.81	0.013
Dual-B	✓		0.090	37.09	14.11	5.52	0.012
Dual-B	✓	✓	**0.089**	**34.06**	**13.82**	**5.00**	**0.011**

The optimal numerical results are highlighted in bold.

**Table 2:** The parameter setting in loss function.

Epoch	1∼25	25∼50	50∼100	1∼25	25∼50	50∼100	1∼25	25∼50	50∼100
λ	1	1	1	1	1	1	1	1	1
$\lambda_{init}$	0	0	0	1	1	1	1	0.5	0
$\lambda_{id}$	0	0	0	0.2	0.2	0.2	0.2	0	0
$\lambda_{dd}$	0	0	0	0.2	0.2	0.2	0.2	0	0
RMSE	0.096			0.095			**0.089**		

The optimal numerical results are highlighted in bold.

**Table 3:** Ablation experiment of dual-attention fusion module.

Conv	Trans	RMSE	MAE	iRMSE	iMAE	REL
✓		0.095	38.71	14.92	5.73	0.013
	✓	0.094	38.80	14.57	5.73	0.013
✓	✓	**0.090**	**37.09**	**14.11**	**5.52**	**0.012**

The optimal numerical results are highlighted in bold.

**Table 4:** The ablation experiments of the Q,K,V configuration in cross-attention in the NYUv2 dataset.

*Q*	*K*	*V*	RMSE	MAE	iRMSE	iMAE	REL
$Q^{G}$	$K^{I}$	$V^{I}$	**0.090**	37.09	**14.11**	5.52	**0.012**
$Q^{I}$	$K^{G}$	$V^{G}$	0.092	37.15	14.25	**5.37**	**0.012**
$Q^{G}$	$K^{G}$	$V^{I}$	0.092	**36.71**	14.29	**5.37**	**0.012**

The optimal numerical results are highlighted in bold.

**Table 5:** Quantitative result on NYUv2 dataset.

Methods	RMSE ↓	REL ↓	$\delta_{1.25}$↑	$\delta_{1.25^{2}}$↑	$\delta_{1.25^{3}}$↑
CSPN [23]	0.115	0.022	99.2	99.9	100.0
Deeplidar [20]	0.115	0.022	99.3	99.9	100.0
DepthNormal [30]	0.112	0.018	99.5	99.9	100.0
FCFR-net [54]	0.106	0.015	99.5	99.9	100.0
ACMNet [55]	0.105	0.015	99.4	99.9	100.0
RGBD-FusionGAN [56]	0.103	0.016	99.4	99.9	100.0
GuideNet [45]	0.101	0.015	99.5	99.9	100.0
CostDCNet [57]	0.096	0.013	99.5	99.9	100.0
NLSPN [25]	0.092	0.012	99.6	99.9	100.0
TWISE [29]	0.092	0.012	99.6	99.9	100.0
LRRU [28]	0.091	0.011	99.6	99.9	100.0
RigNet [47]	0.090	0.013	99.6	99.9	100.0
DySPN [26]	0.090	0.012	99.6	99.9	100.0
GraphCSPN [27]	0.090	0.012	99.6	99.9	-
CompletionFormer [40]	0.090	0.012	-	-	-
BEVDC [58]	**0.089**	0.012	99.6	99.9	100.0
PointDC [32]	**0.089**	0.012	99.6	99.9	100.0
Ours*	0.090	0.012	**99.6**	**99.9**	**100.0**
Ours	**0.089**	**0.011**	**99.6**	**99.9**	**100.0**

↓ indicates that a lower value is better, while ↑ indicates that a higher value is better. The optimal numerical results are highlighted in bold.

**Table 6:** Quantitative result on SUN-RGBD dataset.

Methods	RMSE ↓	MAE ↓	REL ↓	SSIM ↑	$\delta_{1.05}$↑
CSPN [23]	0.116	0.039	0.027	97.9	91.4
Sparse2Dense [16]	0.118	0.040	0.027	98.0	90.7
FusionNet [48]	0.129	0.025	0.020	78.9	94.7
GuideNet [45]	0.088	0.027	0.018	75.3	93.9
DM-LRN [59]	0.098	0.027	0.017	98.8	94.8
FCFR-Net [54]	0.088	0.027	0.020	98.9	95.0
TS-Net [60]	0.086	0.024	0.019	**99.1**	95.8
Ours	**0.082**	**0.020**	**0.016**	98.0	**96.4**

↓ indicates that a lower value is better, while ↑ indicates that a higher value is better. The optimal numerical results are highlighted in bold.

**Table 7:** Inference efficiency of the models.

Models	Inference Time	Inference Memory	Model Size	RMSE
Single-B	0.073 s	2444 M	177 M	0.108
Dual-B	0.166 s	2830 M	468 M	0.090
Dual-B + NLSPN	0.191 s	3138 M	469 M	0.089

## Data Availability

Data is contained within the article.

## Abbreviations

The following abbreviations are used in this manuscript:

SPN	Spatial Propagation Network
CNN	Convolutional Neural Networks
NLSPN	Non-Local Spatial Propagation Network
BN	Batch Normalization

ViT	Vision Transformer
JCAT	Joint Convolutional Attention and Transformer
DAFM	Dual-Attention Fusion Module
LN	Layer Normalization
LP	Layer Projection
CA	Channel Attention
SA	Spatial Attention
RMSE	Root Mean Square Error
MAE	Mean Absolute Error
iRMSE	Inverse Root Mean Square Error
iMAE	Inverse Mean Absolute Error
Rel	Absolute Relative Error
SSIM	Structural Similarity Index Measure
